# CABS-flex predictions of protein flexibility compared with NMR ensembles

**DOI:** 10.1093/bioinformatics/btu184

**Published:** 2014-05-02

**Authors:** Michal Jamroz, Andrzej Kolinski, Sebastian Kmiecik

**Affiliations:** Laboratory of Theory of Biopolymers, Faculty of Chemistry, University of Warsaw, Pasteura 1, Warsaw 02-093, Poland

## Abstract

**Motivation:** Identification of flexible regions of protein structures is important for understanding of their biological functions. Recently, we have developed a fast approach for predicting protein structure fluctuations from a single protein model: the CABS-flex. CABS-flex was shown to be an efficient alternative to conventional all-atom molecular dynamics (MD). In this work, we evaluate CABS-flex and MD predictions by comparison with protein structural variations within NMR ensembles.

**Results:** Based on a benchmark set of 140 proteins, we show that the relative fluctuations of protein residues obtained from CABS-flex are well correlated to those of NMR ensembles. On average, this correlation is stronger than that between MD and NMR ensembles. In conclusion, CABS-flex is useful and complementary to MD in predicting protein regions that undergo conformational changes as well as the extent of such changes.

**Availability and implementation:** The CABS-flex is freely available to all users at http://biocomp.chem.uw.edu.pl/CABSflex.

**Contact**: sekmi@chem.uw.edu.pl

**Supplementary information:** Supplementary data are available at *Bioinformatics* online.

## 1 INTRODUCTION

Proteins exist in solution as ensembles of structurally different conformational states. These ensembles can exhibit different degrees of structural diversity, ranging from almost static to highly mobile protein regions. Structural flexibility is one of the key characteristics of proteins and allows them to play important functional roles in living organisms. Thus, knowledge of conformational states in native-state ensembles can provide important insights into protein functions (e.g. molecular recognition, protein allostery) (Fenwick *et al.*, 2011; Gerek *et al.*, 2013; Hilser, 2010; Wrabl *et al.*, 2011) as well as protein evolution (Gerek *et al.*, 2013; Wrabl *et al.*, 2011).

Most of the known protein structures have been solved by X-ray crystallography and deposited in the Protein Data Bank (PDB) as a single model. A single crystal structure, however, gives little information about conformational heterogeneity or model accuracy, and this is why the crystallographic community has been urged to deposit an ensemble of solutions whenever possible (Furnham *et al.*, 2006). An ensemble view of protein structures comes predominantly from NMR spectroscopy, which is the method of choice for the determination of protein structure and dynamics in solution (Markwick *et al.*, 2008). NMR spectroscopy routinely provides an ensemble of protein models, which usually consists of 20 conformers on average. The precision and accuracy of NMR ensembles have been a subject of a long-standing dispute in the field (Spronk *et al.*, 2003). The structure diversity of NMR-derived ensembles may depend not only on the quality and amount of collected data but also on the computational procedures used for generating and selecting low-energy models that fit experimental data. Nevertheless, it has been demonstrated that NMR ensembles may provide valuable insights into protein flexibility that is of practical use in structure-to-function studies (Bolstad and Anderson, 2008; Damm and Carlson, 2007; Isvoran *et al.*, 2011; Knegtel *et al.*, 1997). Among these studies, particularly interesting is probably the first comparison of NMR ensembles and a collection of crystal structures from the point of using them in structure-based drug design, performed by Damm and Carlson (2007). They demonstrated that for human immunodeficiency virus-1 protease (hiv-1p), there is more structural variation between 28 structures in an NMR ensemble than between 90 crystal structures bound to a variety of ligands. Because the NMR ensemble-derived model provided the most general, yet accurate, representation of the active site of hiv-1p, the authors strongly encourage the use of NMR models in structure-based drug design.

Except for experimental sources, the present views on protein flexibility have been largely obtained, thanks to the use of molecular dynamics (MD). In the past decades, MD has become an indispensable tool for determining conformationally heterogeneous states of proteins, most often through unbiased simulations starting from experimental static structures or in combination with experimental data (Fisette *et al.*, 2012; Vendruscolo, 2007). The idea that unbiased MD simulations capture the true dynamic nature of proteins was supported by a study showing that various MD force-fields provide a consensus picture of protein fluctuations in solution (Rueda *et al.*, 2007). Using the MD simulation data from this study, we recently demonstrated that the structural and dynamics characteristics of MD trajectories are fairly consistent with simulation results from a coarse-grained protein model—the CABS model (Jamroz *et al.*, 2013b). Importantly, the computational cost of obtaining near-native dynamics by CABS simulations was proved to be much lower (∼6 × 10^3^ times) than that of MD [technically, this is the cost of achieving a residue fluctuation profile that best fits that obtained from 10 ns MD simulations, see details in Jamroz *et al.* (2013b)]. Following this work, we implemented the developed CABS-model-based protocol for fast simulations of near-native dynamics in a web server called CABS-flex (Jamroz *et al.*, 2013a).

In previous works, we compared CABS-flex predictions of protein flexibility with a large set of MD simulation data (Jamroz *et al.*, 2013a, b). The comparison tests showed that the CABS-flex method is a computationally efficient alternative to MD. The present work describes a comparison of protein fluctuations obtained from CABS-flex and MD simulations with fluctuations derived from NMR ensembles.

## 2 METHODS

### 2.1 Benchmark set

We used a protein benchmark set constructed and reported by Jamroz *et al.* (2012). The benchmark set contains 140 non-redundant proteins determined by NMR (with NMR ensembles consisting of >10 models in their PDB files) and MD simulation trajectories deposited in the MoDEL database (Meyer *et al.*, 2010). The protein set is non-redundant in the sense that it contains no two proteins that have sequence identity higher than a 35% cutoff according to the pisces database (Wang and Dunbrack, 2003).

### 2.2 CABS-flex method

The CABS-flex method follows our earlier work (Jamroz *et al.*, 2013b) where we demonstrated that the consensus view of protein near-native dynamics obtained from 10 ns MD simulations (all-atom, explicit water, using the four most popular force-fields for all protein metafolds) is consistent with dynamics from the CABS model. The CABS-flex simulation length has been optimized to obtain the best possible convergence with the 10 ns MD simulations [see details in Jamroz *et al.* (2013b)].

CABS is a well-established coarse-grained protein modeling tool for predicting protein dynamics (Kmiecik and Kolinski, 2007, 2011; Kmiecik *et al.*, 2012) and protein structure (Blaszczyk *et al.*, 2013; Kmiecik *et al.*, 2007; Kolinski and Bujnicki, 2005). The CABS design is a compromise between high sampling efficiency and high resolution of protein representation. The CABS protein representation is reduced to up to four pseudo-atoms per residue, the force field uses knowledge-based potentials (accounting for solvent effects in an implicit fashion), and the sampling is realized by the Monte Carlo method [details are given in Kolinski (2004)]. The resolution of CABS-generated models allows the reconstruction of physically sound atomistic models (Kmiecik *et al.*, 2007, 2012; Wabik *et al.*, 2013).

The CABS-based procedure for the simulation of near-native dynamics has been made available as a CABS-flex web server (Jamroz *et al.*, 2013a). The CABS-flex server requires input of a single protein structure and outputs a residue fluctuation profile together with accompanying analysis. Additionally, the CABS-flex pipeline incorporates multiscale reconstruction and optimization procedures (Gront *et al.*, 2012; Kmiecik *et al.*, 2011), which output an ensemble of protein models (in all-atom resolution) reflecting the flexibility of the input structure.

### 2.3 Computing residue fluctuation profiles

Based on the generated trajectory (CABS-flex or MD) or NMR ensemble, superimposed with theseus (Theobald and Wuttke, 2006), a residue-fluctuation profile (root mean square fluctuation, RMSF), is calculated as follows:

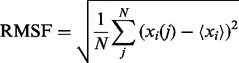

where <> denotes the average over the whole NMR ensemble or trajectory, and *x* is the position of residue (Cα atom) *i* in the trajectory or NMR ensemble model *j*.

For the comparison of residue fluctuation profiles obtained from CABS-flex, MD and NMR ensembles, we used Spearman’s rank correlation coefficient. It quantifies the extent of statistical dependence between pairs of observations (and is better suited to reflect data correlation in the presence of outlier values than the Pearson correlation coefficient). Spearman’s rank correlation was also used in our earlier comparisons of MD and CABS-flex fluctuation profiles to which we refer in this study (Jamroz *et al.*, 2013a, b).

Note that the statistical errors of RMSF values generated by CABS-flex are reflected in root mean squared deviations (RMSD) between RMSF profile data (Fig. 2B).

## 3 RESULTS

In this work, we used a benchmark protein set of 140 proteins collected and reported by Jamroz *et al.* (2012).

**Fig. 1.**
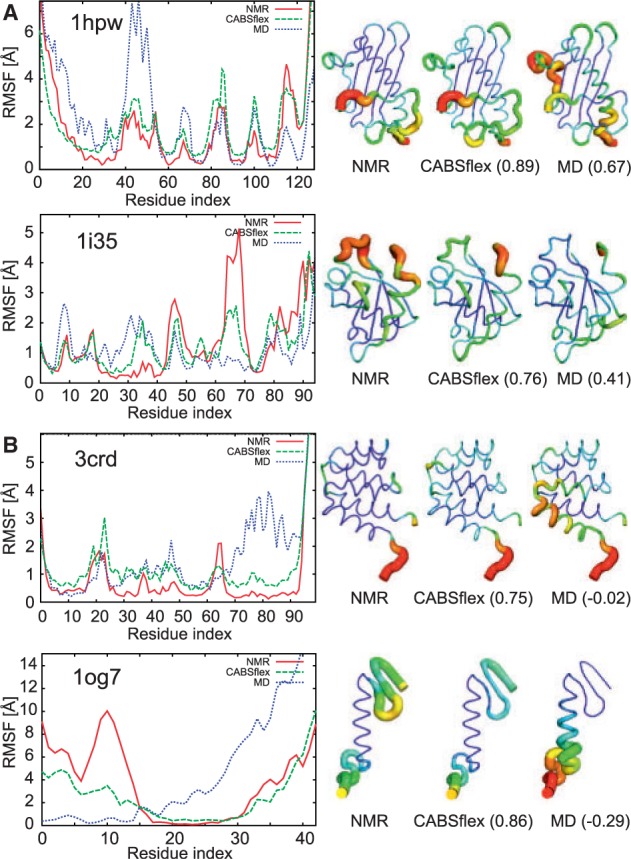
Comparison of residue-fluctuation profiles for example proteins from the benchmark set. The presented examples illustrate several levels of prediction accuracy in comparison with NMR ensembles: (**A**) high by CABS-flex and average or below average by MD, (**B**) high by CABS-flex and poor by MD. For each protein, residue-fluctuation profiles are visualized on a plot and projected on protein models. The plots present RMSF values (in Ångstroms) derived from NMR ensembles (red line) and simulation trajectories: CABS (green line) and MD (blue line). The RMSF values are also visualized in the respectively signed protein models (in brackets: correlation coefficients for residue fluctuations between NMR and CABS-flex or MD). In the protein models, colors and tube thickness denote RMSF values scaled from the maximum (red color, thick tube) to minimum (blue color, thin tube). Analogous plots for the entire test set are presented in Supplementary Figure S1

In Figure 1, we show a comparison of flexibility for four example proteins from the benchmark set. Structural flexibility is presented in the figure as residue-fluctuation profiles, i.e. RMSF values for each residue (see Section 2.3), visualized in plots or projected on protein models.

**Fig. 2.**
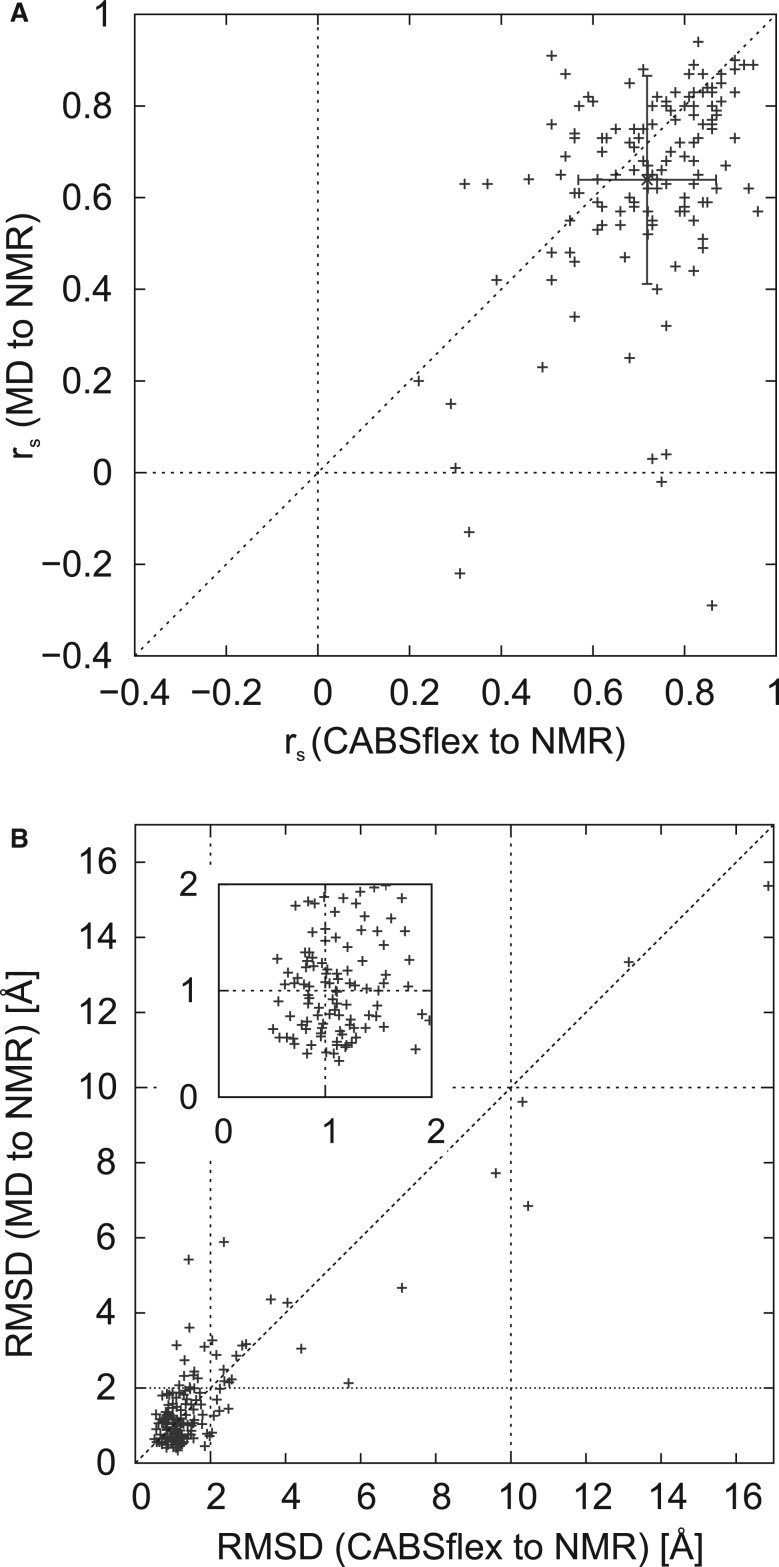
Comparison of residue-fluctuation profiles for the benchmark set. For the set of 140 protein structures, a comparison between CABS-flex and NMR is presented together with that of MD and NMR. For each protein, residue-fluctuation profiles (root mean squared fluctuations, RMSF) are compared using (**A**) Spearman’s correlation coefficient (*r_s_*) and (**B**) average RMSD (root-mean square deviation) values

In Figure 2, we present a comparison of residue-fluctuation profiles for the entire benchmark set. The comparison is done using Spearman’s correlation coefficient (*r_s_*) (Fig. 2A) and average root mean square deviation (RMSD) between RMSF values of MD/NMR/CABS-flex (Fig. 2B). Remarkably, the average *r_s_* between CABS-flex and NMR ensembles is slightly less scattered than that between MD and NMR: 0.72 (±0.15) and 0.64 (±0.23), respectively (standard deviation values are given in brackets).

The *r_s_* correlation coefficient is a measure of statistical dependence between compared residue-fluctuation profiles and does not reflect differences in profile amplitudes. This is reflected in the average RMSD between the compared profiles shown in Figure 2B. As presented in the plot, the RMSD between NMR profiles and CABS-flex or MD profiles usually does not exceed 2 Å. In general, the higher the structural heterogeneity in NMR ensembles, the higher is the presented RMSD values. The largest RMSD values correspond to proteins with highly flexible regions. For instance, the highest RMSD values (NMR to MD as well as CABS-flex to NMR) correspond to the structure of cide-N Domain of cide-B protein (PDB ID: 1d4b), which has largely disordered regions of substantial length (residues 1–31 and 111–122). The exact *r_s_* and RMSD values for each protein are given in Supplementary Table S1 together with accompanying data.

## 4 DISCUSSION

The proteins from the benchmark set represent different degrees of structural variability within NMR ensembles. The degree of variability (average displacement per residue) ranges from 0.2 to almost 12 Å. For the entire benchmark set, the average displacement per residue in NMR ensembles is 1.68 Å (the values for each protein are given in Supplementary Table S1).

The analysis of variability of NMR ensembles versus prediction quality showed a tendency that the higher the flexibility observed in an NMR ensemble, the better the correlation coefficient (*r_s_*) between NMR and CABS-flex or MD fluctuation profiles. For 57% of proteins from the benchmark set, the average displacement within their NMR ensembles is >1 Å. In this subset, the average *r_s_* between NMR and simulation (CABS-flex or MD) is slightly higher (0.78 for CABS-flex and 0.69 for MD) than for proteins with less variable NMR ensembles (Table 1).

**Table 1.** Average Spearman’s correlation coefficients (*r_s_*) between residue-fluctuation profiles

Compared methods	Benchmark dataset of NMR-solved proteins		
	Entire dataset (140 proteins)	Subset with RMSD of NMR ensemble ≤1 Å (60 proteins)	Subset with RMSD of NMR ensemble >1 Å (80 proteins)
CABS-flex versus NMR	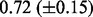	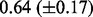	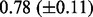
MD versus NMR	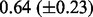	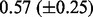	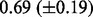
CABS-flex versus MD	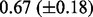	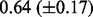	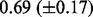

*Note*: The table shows an average pairwise comparison between CABS-flex, MD and NMR ensembles. The average correlation values (and standard deviations in brackets) are presented for the entire protein benchmark set and its subsets having average fluctuations in the NMR ensemble: lower (RMSD ≤ 1 Å) or higher (RMSD >1 Å).

Furthermore, we examined another subset of proteins for which CABS-flex predictions were the poorest (with *r_s_* < 0.5: 1k8b, 1waz, 1kkg, 1k5k, 1cok, 1sgg, 1pcp, 1pav, 1p6q, 2rgf). In this subset of 10 proteins, the average *r_s_* between NMR and CABS-flex fluctuation profiles was 0.35, while that between NMR and MD was even lower: 0.26. The subset analysis showed that 9 of 10 proteins had NMR ensembles exhibiting almost no or small flexibility, in contrast to CABS-flex or MD predictions (the exception was 1pcp, which has a small amount of secondary structure only). For these nine proteins, the average displacement per residue within NMR ensembles was below 0.5 Å (counted for the entire or most of the chain). Such large rigidity does not seem to be justified by the structural characteristics of these proteins. For at least some of them, highly homologous counterparts can be found in the PDB, which show more structural variation than the analyzed NMR ensembles.

The above observations suggest that an important source of poor correspondence between fluctuations from computational predictions (from CABS-flex or MD) and NMR ensembles is the underestimation of fluctuations in NMR ensembles. Several studies strongly indicate that fluctuations in NMR ensembles are underestimated and do not reflect real structural heterogeneity (Pfeiffer *et al.*, 1997; Scheek *et al.*, 1995; Spronk *et al.*, 2003; Torda *et al.*, 1990). The underestimations are largely due to shortcomings of computational procedures used to generate the ensembles based on NMR data.

The CABS-flex method provides an alternative to other efficient computational tools generating protein residue fluctuation profiles, such as sequence-based predictors of protein disordered regions (Mészáros *et al.*, 2014) or coarse-grained normal mode analysis (NMA; Ma, 2005). Most disorder prediction algorithms [such as DISOPRED, Ward *et al.* (2004)] perform well for stable globular domains or highly flexible disordered regions without a strong structural preference. However, their performance does not meet expectations for structurally ambiguous regions (Mészáros *et al.*, 2014). Therefore, in comparison with sequence-based disorder prediction algorithms, CABS-flex is better suited to detecting non-obvious dynamic behavior (e.g. significant fluctuations within the well-defined secondary structural elements that could be of biological importance). Another class of commonly used algorithms that compute protein fluctuation profiles use NMA based on elastic network models or other coarse-grained models [e.g. WEBnma server, Hollup *et al.* (2005)]. In comparison with elastic network models, CABS-flex uses more detailed information on the protein system and generates residue fluctuation profiles better correlated (on average) with those obtained by all-atom MD [see our Section 4 in Jamroz *et al.* (2013b)]. The CABS-flex-generated models (or trajectory) can also be subjected to NMA. As we demonstrated earlier (Jamroz *et al.*, 2013b), essential movements derived from CABS-flex trajectories might not be accurate individually, but when considered together they provide a similar description to that obtained by all-atom MD. Readers interested in applying the NMA may refer to a review on the usefulness and limitations of the method (Ma, 2005).

## 5 CONCLUSION

Due to the dynamic nature of proteins, structure-based studies of protein functions require accurate description of protein flexibility.

Crystallographic B-factors are perhaps the most common measure used for the elucidation of residue fluctuations, and this is probably because the majority of known structures have been solved by X-ray crystallography. The B-factors reflect protein flexibility but are also influenced by crystallization conditions, the refinement method (used for the interpretation of X-ray data) and, importantly, the molecular environment of the crystal structure. The crystal environment has a significant effect on protein flexibility: the spectrum of fluctuations is considerably flattened in crystal as compared with that in solution (Eastman *et al.*, 1999). Moreover, most X-ray structures have been determined at cryogenic temperatures. Crystal cryo-cooling has been shown to reduce B-factors, introduce packing defects and it may result in unrealistically unique non-functional structures (Fraser *et al.*, 2011; Rasmussen *et al.*, 1992). Therefore, descriptions of protein flexibility derived from X-ray models and B-factors must be approached with caution.

NMR and all-atom MD are now the methods of choice for investigation of protein flexibility in solution. Because of the difficulty of NMR studies and timescale problems in all-atom MD, coarse-grained methods have emerged as an inexpensive and powerful alternative. The design of coarse-grained methods successfully applied for large timescale investigations of protein dynamics encompasses entirely different modeling strategies (Emperador *et al.*, 2008; Jamroz *et al.*, 2013b; Maisuradze *et al.*, 2010). An excellent review on the successes and shortcomings of diverse coarse-grained representations of protein flexibility is provided in (Orozco *et al.*, 2011).

In this work, we compare CABS-flex predictions of protein fluctuations with that derived from NMR ensembles and MD simulations. The comparison shows that CABS-flex produces, on average, a more similar distribution of residue fluctuations to NMR ensembles than MD does. This is due to more efficient sampling compared with MD, which leads to additional fluctuations or fluctuation amplitudes that better fit the NMR ensemble data. Moreover, the results from CABS-flex and MD can complement each other in the sense that the flexibility of some protein regions may be better retrieved by one of these methods, while the remaining part by the other one. In summary, our results suggest that for the accurate assessment of protein flexibility it is reasonable to analyze results from both CABS-flex and atomic MD simulations. Because the CABS-flex method provides a significantly cheaper means of accessing backbone dynamics than atomic MD, it is a promising tool for larger and/or initial reconnaissance screening studies, for example, of the effect of mutations on protein stability or structure-based drug design.

*Funding*: Foundation for Polish Science TEAM project [TEAM/2011-7/6] co-financed by the EU European Regional Development Fund operated within the Innovative Economy Operational Program; Polish National Science Centre [NN301071140]; Polish Ministry of Science and Higher Education [IP2011 024371].

*Conflict of Interest*: none declared.

## Supplementary Material

Supplementary Data
