# Supplementary material for: CABS-flex predictions of protein flexibility compared with NMR ensembles
Source: Bioinformatics. 2014 May 2;30(15):2150–4. doi: 10.1093/bioinformatics/btu184 (PMC4103595; doi:10.1093/bioinformatics/btu184)
Supplement: Supplementary Data [file supp_30_15_2150__index.html]

CABS-flex predictions of protein flexibility compared with NMR ensembles — CABS-flex predictions of protein flexibility compared with NMR ensembles — CABS-flex predictions of protein flexibility compared with NMR ensembles — Supplementary Data 

# CABS-flex predictions of protein flexibility compared with NMR ensembles

## Supplementary Data

files

**Files in this Data Supplement:**

- Supplementary Data - docx file
