# Supplementary material for: CABS-flex predictions of protein flexibility compared with NMR ensembles
Source: Bioinformatics. 2014 May 2;30(15):2150–4. doi: 10.1093/bioinformatics/btu184 (PMC4103595; doi:10.1093/bioinformatics/btu184)

**Supplementary info**

**Table S1.** Comparison of residue-fluctuation profiles for the entire benchmark set of 140 proteins. For each protein, residue-fluctuation profiles (root mean-square fluctuations, RMSF) are compared using: Spearman’s correlation coefficient (r_s_), and RMSD (root-mean square deviation) values. The comparison is presented for CABS-flex vs. NMR, MD vs. NMR and CABS-flex vs. MD fluctuations profiles. Additionally, average fluctuations (RMSF) per residue (in Ångstroms) for NMR, CABS-flex and MD fluctuations profiles are given.

| # | PDB ID | Number of protein residues | CABS-flex vs. NMR | | MD vs. NMR | | CABS-flex vs. MD | | Average fluctuations (RMSF) per residue (in Ångstroms) | | |
| --- | --- | --- | --- | --- | --- | --- | --- | --- | --- | --- | --- |
|  |  |  | r_s_ | RMSD | r_s_ | RMSD | r_s_ | RMSD | NMR | CABS-flex | MD |
| 1 | 1a9v | 129 | 0.57 | 1.23 | 0.80 | 0.68 | 0.72 | 0.79 | 0.59 | 1.60 | 1.06 |
| 2 | 1adn | 92 | 0.95 | 2.50 | 0.89 | 2.15 | 0.91 | 1.27 | 3.78 | 3.49 | 2.76 |
| 3 | 1ah9 | 71 | 0.86 | 1.02 | 0.77 | 1.20 | 0.80 | 1.08 | 1.04 | 1.47 | 0.83 |
| 4 | 1apq | 53 | 0.82 | 2.16 | 0.63 | 2.88 | 0.51 | 1.46 | 2.80 | 2.41 | 1.61 |
| 5 | 1b75 | 94 | 0.88 | 0.70 | 0.87 | 0.55 | 0.87 | 0.54 | 0.92 | 1.43 | 1.14 |
| 6 | 1ba5 | 53 | 0.68 | 1.48 | 0.60 | 0.76 | 0.78 | 1.13 | 1.19 | 1.64 | 1.09 |
| 7 | 1bak | 119 | 0.61 | 1.33 | 0.59 | 1.93 | 0.75 | 1.48 | 1.83 | 2.00 | 1.95 |
| 8 | 1be2 | 91 | 0.51 | 0.83 | 0.42 | 0.41 | 0.49 | 0.69 | 0.60 | 1.22 | 0.82 |
| 9 | 1c3y | 108 | 0.84 | 1.84 | 0.51 | 3.10 | 0.52 | 2.57 | 1.60 | 2.09 | 3.37 |
| 10 | 1cis | 66 | 0.73 | 1.09 | 0.55 | 0.82 | 0.52 | 0.86 | 0.63 | 1.56 | 1.12 |
| 11 | 1ckv | 141 | 0.84 | 10.46 | 0.87 | 6.85 | 0.82 | 4.24 | 7.17 | 3.48 | 4.28 |
| 12 | 1co1 | 115 | 0.73 | 1.00 | 0.54 | 1.47 | 0.60 | 1.29 | 1.94 | 1.71 | 1.09 |
| 13 | 1co4 | 42 | 0.94 | 0.88 | 0.62 | 1.31 | 0.66 | 1.43 | 2.06 | 2.74 | 1.66 |
| 14 | 1cok | 68 | 0.32 | 1.35 | 0.63 | 1.28 | 0.69 | 1.39 | 0.99 | 1.86 | 0.92 |
| 15 | 1cou | 85 | 0.88 | 2.95 | 0.85 | 3.17 | 0.76 | 0.55 | 3.39 | 1.50 | 1.26 |
| 16 | 1cqu | 56 | 0.91 | 1.98 | 0.83 | 0.72 | 0.91 | 1.98 | 1.14 | 2.69 | 1.28 |
| 17 | 1ctl | 85 | 0.87 | 2.05 | 0.79 | 3.27 | 0.70 | 1.90 | 4.20 | 4.14 | 3.02 |
| 18 | 1d2b | 126 | 0.86 | 1.10 | 0.83 | 1.00 | 0.76 | 0.88 | 0.68 | 1.61 | 1.22 |
| 19 | 1d4b | 122 | 0.86 | 16.96 | 0.84 | 15.37 | 0.84 | 2.43 | 10.31 | 3.21 | 3.05 |
| 20 | 1d8b | 81 | 0.74 | 1.01 | 0.82 | 0.42 | 0.79 | 0.92 | 0.68 | 1.42 | 0.86 |
| 21 | 1dro | 122 | 0.84 | 1.21 | 0.76 | 1.41 | 0.83 | 1.34 | 1.48 | 2.06 | 1.60 |
| 22 | 1ds9 | 198 | 0.55 | 1.17 | 0.48 | 2.07 | 0.76 | 1.36 | 0.62 | 1.58 | 1.80 |
| 23 | 1e2b | 106 | 0.69 | 0.57 | 0.71 | 0.56 | 0.74 | 0.70 | 0.87 | 1.24 | 1.01 |
| 24 | 1egl | 70 | 0.80 | 1.01 | 0.60 | 1.16 | 0.47 | 1.52 | 1.32 | 2.15 | 1.02 |
| 25 | 1egx | 115 | 0.86 | 1.16 | 0.76 | 0.59 | 0.72 | 0.77 | 0.35 | 1.30 | 0.78 |
| 26 | 1eik | 77 | 0.83 | 1.62 | 0.65 | 1.68 | 0.55 | 1.07 | 1.53 | 2.16 | 1.81 |
| 27 | 1eiw | 111 | 0.66 | 0.96 | 0.54 | 0.57 | 0.29 | 1.13 | 0.98 | 1.76 | 1.02 |
| 28 | 1eo0 | 77 | 0.51 | 0.90 | 0.48 | 1.82 | 0.05 | 1.23 | 0.21 | 1.04 | 1.74 |
| 29 | 1eww | 90 | 0.73 | 1.00 | 0.76 | 1.58 | 0.68 | 1.20 | 0.77 | 1.57 | 1.73 |
| 30 | 1ey1 | 139 | 0.80 | 1.09 | 0.57 | 1.74 | 0.66 | 1.36 | 0.97 | 1.36 | 2.03 |
| 31 | 1fsb | 40 | 0.86 | 1.11 | 0.80 | 0.52 | 0.54 | 1.14 | 0.86 | 1.86 | 1.01 |
| 32 | 1g84 | 105 | 0.82 | 1.11 | 0.83 | 0.99 | 0.83 | 0.96 | 0.93 | 1.53 | 1.25 |
| 33 | 1ghc | 75 | 0.72 | 0.62 | 0.57 | 1.06 | 0.77 | 0.88 | 1.52 | 1.45 | 1.83 |
| 34 | 1gjx | 81 | 0.56 | 0.96 | 0.61 | 0.60 | 0.80 | 0.58 | 0.60 | 1.45 | 1.05 |
| 35 | 1gk5 | 49 | 0.69 | 2.25 | 0.59 | 1.98 | 0.81 | 1.31 | 0.89 | 2.85 | 2.17 |
| 36 | 1gnc | 175 | 0.80 | 1.45 | 0.58 | 2.02 | 0.66 | 1.16 | 2.25 | 1.98 | 1.90 |
| 37 | 1gxi | 73 | 0.93 | 1.49 | 0.89 | 0.86 | 0.87 | 2.06 | 1.42 | 2.50 | 1.10 |
| 38 | 1gyf | 62 | 0.71 | 1.20 | 0.88 | 0.49 | 0.81 | 0.80 | 0.43 | 1.42 | 0.79 |
| 39 | 1h5p | 95 | 0.91 | 1.07 | 0.73 | 0.92 | 0.80 | 1.40 | 1.01 | 1.92 | 1.00 |
| 40 | 1hpw | 129 | 0.89 | 0.84 | 0.67 | 1.84 | 0.62 | 1.80 | 1.74 | 1.97 | 2.26 |
| 41 | 1hqi | 90 | 0.76 | 1.57 | 0.32 | 1.99 | 0.23 | 1.27 | 2.78 | 1.67 | 1.78 |
| 42 | 1hra | 80 | 0.56 | 1.17 | 0.46 | 1.87 | 0.56 | 1.47 | 0.75 | 1.60 | 1.98 |
| 43 | 1hy9 | 41 | 0.82 | 1.34 | 0.44 | 1.57 | 0.69 | 0.72 | 0.77 | 1.92 | 1.81 |
| 44 | 1i2u | 44 | 0.57 | 1.50 | 0.61 | 1.00 | 0.72 | 0.82 | 0.41 | 1.51 | 1.20 |
| 45 | 1i35 | 95 | 0.76 | 0.83 | 0.04 | 1.28 | 0.41 | 0.75 | 1.37 | 1.22 | 1.10 |
| 46 | 1iba | 78 | 0.74 | 0.99 | 0.64 | 1.88 | 0.75 | 1.36 | 0.68 | 1.49 | 1.67 |
| 47 | 1ica | 40 | 0.83 | 2.04 | 0.73 | 0.81 | 0.82 | 2.56 | 1.35 | 2.82 | 1.08 |
| 48 | 1iez | 217 | 0.74 | 2.37 | 0.62 | 2.18 | 0.63 | 1.04 | 2.72 | 1.51 | 1.70 |
| 49 | 1ify | 49 | 0.56 | 1.23 | 0.73 | 1.07 | 0.60 | 1.00 | 0.65 | 1.34 | 1.38 |
| 50 | 1ig6 | 107 | 0.72 | 1.55 | 0.67 | 2.34 | 0.58 | 1.22 | 1.87 | 2.10 | 1.77 |
| 51 | 1ioj | 57 | 0.82 | 9.60 | 0.55 | 7.72 | 0.51 | 3.82 | 8.80 | 2.13 | 5.32 |
| 52 | 1iq3 | 110 | 0.91 | 1.44 | 0.90 | 3.61 | 0.84 | 2.54 | 3.58 | 3.26 | 2.24 |
| 53 | 1irz | 64 | 0.76 | 1.85 | 0.68 | 0.45 | 0.90 | 1.89 | 1.36 | 2.26 | 1.29 |
| 54 | 1iuy | 92 | 0.87 | 5.68 | 0.78 | 2.13 | 0.85 | 3.82 | 4.12 | 2.18 | 3.25 |
| 55 | 1iv0 | 98 | 0.76 | 2.09 | 0.80 | 1.26 | 0.52 | 1.27 | 1.72 | 1.65 | 1.38 |
| 56 | 1ivt | 122 | 0.66 | 1.11 | 0.57 | 0.88 | 0.84 | 0.72 | 0.72 | 1.63 | 1.18 |
| 57 | 1iym | 55 | 0.73 | 1.28 | 0.65 | 1.05 | 0.64 | 0.92 | 0.66 | 1.82 | 1.49 |
| 58 | 1j0t | 78 | 0.56 | 1.12 | 0.34 | 1.11 | 0.55 | 0.81 | 0.53 | 1.52 | 1.27 |
| 59 | 1j3g | 187 | 0.73 | 1.75 | 0.65 | 1.56 | 0.84 | 1.60 | 0.69 | 2.02 | 1.39 |
| 60 | 1jbj | 186 | 0.59 | 4.05 | 0.82 | 4.27 | 0.77 | 0.97 | 3.14 | 1.92 | 1.29 |
| 61 | 1jt8 | 102 | 0.82 | 1.66 | 0.78 | 2.26 | 0.84 | 3.43 | 2.85 | 2.51 | 3.26 |
| 62 | 1jw2 | 72 | 0.62 | 0.72 | 0.73 | 1.80 | 0.76 | 1.85 | 1.06 | 1.41 | 2.01 |
| 63 | 1jw3 | 140 | 0.68 | 0.71 | 0.72 | 0.50 | 0.68 | 0.83 | 0.93 | 1.33 | 0.95 |
| 64 | 1k0h | 117 | 0.82 | 7.10 | 0.89 | 4.67 | 0.72 | 3.04 | 4.89 | 2.79 | 3.09 |
| 65 | 1k5k | 87 | 0.31 | 1.72 | -0.22 | 1.87 | 0.12 | 1.60 | 0.23 | 1.86 | 1.42 |
| 66 | 1k8b | 52 | 0.22 | 0.85 | 0.20 | 1.36 | 0.73 | 0.78 | 0.39 | 1.04 | 1.43 |
| 67 | 1k8h | 133 | 0.83 | 0.78 | 0.94 | 0.68 | 0.81 | 0.89 | 1.37 | 1.80 | 1.52 |
| 68 | 1kkg | 108 | 0.30 | 1.29 | 0.01 | 1.82 | 0.46 | 1.32 | 0.40 | 1.50 | 1.52 |
| 69 | 1kvi | 79 | 0.69 | 0.94 | 0.58 | 0.84 | 0.51 | 1.27 | 1.03 | 1.63 | 1.30 |
| 70 | 1l7b | 92 | 0.67 | 1.55 | 0.47 | 1.43 | 0.82 | 1.24 | 1.07 | 2.28 | 1.75 |
| 71 | 1lkn | 89 | 0.56 | 1.19 | 0.74 | 0.47 | 0.75 | 0.85 | 0.41 | 1.29 | 0.71 |
| 72 | 1lq7 | 67 | 0.72 | 0.82 | 0.62 | 1.22 | 0.86 | 0.67 | 0.35 | 0.98 | 1.32 |
| 73 | 1ly7 | 121 | 0.63 | 1.25 | 0.73 | 0.51 | 0.68 | 1.00 | 0.61 | 1.67 | 0.85 |
| 74 | 1m58 | 106 | 0.78 | 1.13 | 0.83 | 0.34 | 0.77 | 0.96 | 0.65 | 1.54 | 0.80 |
| 75 | 1mg8 | 78 | 0.61 | 1.08 | 0.53 | 0.41 | 0.66 | 0.83 | 0.60 | 1.41 | 0.85 |
| 76 | 1mk3 | 178 | 0.55 | 2.24 | 0.55 | 1.39 | 0.70 | 1.68 | 1.67 | 2.15 | 1.90 |
| 77 | 1mut | 129 | 0.71 | 0.56 | 0.68 | 0.90 | 0.70 | 0.76 | 1.34 | 1.52 | 1.70 |
| 78 | 1n5h | 105 | 0.77 | 1.24 | 0.70 | 0.73 | 0.83 | 1.06 | 1.04 | 1.86 | 1.04 |
| 79 | 1n6z | 105 | 0.74 | 1.79 | 0.40 | 1.29 | 0.66 | 1.48 | 1.22 | 2.43 | 1.61 |
| 80 | 1n91 | 108 | 0.61 | 1.55 | 0.64 | 1.07 | 0.63 | 1.22 | 0.92 | 2.16 | 1.48 |
| 81 | 1nfa | 178 | 0.84 | 2.85 | 0.76 | 3.13 | 0.84 | 0.99 | 2.49 | 2.07 | 1.95 |
| 82 | 1nso | 107 | 0.81 | 0.88 | 0.82 | 1.55 | 0.71 | 1.37 | 1.54 | 2.15 | 2.52 |
| 83 | 1ny8 | 97 | 0.65 | 1.78 | 0.75 | 1.04 | 0.57 | 1.68 | 1.27 | 1.92 | 1.79 |
| 84 | 1og7 | 43 | 0.86 | 2.36 | -0.29 | 5.89 | -0.02 | 4.88 | 3.76 | 2.58 | 4.59 |
| 85 | 1oqa | 110 | 0.84 | 1.46 | 0.83 | 1.97 | 0.84 | 1.35 | 1.36 | 2.08 | 1.19 |
| 86 | 1ovq | 138 | 0.82 | 1.31 | 0.72 | 2.74 | 0.91 | 1.98 | 2.13 | 2.36 | 1.34 |
| 87 | 1owa | 156 | 0.83 | 13.14 | 0.94 | 13.34 | 0.75 | 3.08 | 11.63 | 5.02 | 4.82 |
| 88 | 1p1a | 85 | 0.69 | 1.14 | 0.75 | 0.62 | 0.70 | 0.87 | 0.96 | 1.78 | 1.17 |
| 89 | 1p68 | 102 | 0.62 | 0.65 | 0.54 | 1.17 | 0.72 | 0.74 | 0.36 | 0.91 | 1.31 |
| 90 | 1p6q | 129 | 0.46 | 1.39 | 0.64 | 1.02 | 0.48 | 1.00 | 0.31 | 1.36 | 1.16 |
| 91 | 1p6r | 82 | 0.80 | 1.00 | 0.69 | 1.08 | 0.76 | 0.84 | 1.32 | 1.46 | 1.09 |
| 92 | 1p9c | 45 | 0.96 | 1.42 | 0.57 | 5.42 | 0.44 | 5.27 | 5.83 | 5.25 | 3.07 |
| 93 | 1pav | 78 | 0.39 | 0.89 | 0.42 | 1.23 | 0.41 | 0.80 | 0.20 | 0.81 | 1.29 |
| 94 | 1pc0 | 61 | 0.84 | 0.74 | 0.49 | 1.12 | 0.60 | 0.87 | 0.78 | 1.26 | 1.27 |
| 95 | 1pcp | 106 | 0.37 | 1.11 | 0.63 | 1.16 | 0.51 | 1.12 | 1.79 | 2.11 | 2.42 |
| 96 | 1pfj | 108 | 0.68 | 0.97 | 0.85 | 0.65 | 0.64 | 0.91 | 1.00 | 1.66 | 1.27 |
| 97 | 1plo | 122 | 0.76 | 1.04 | 0.81 | 1.07 | 0.74 | 1.62 | 1.52 | 2.26 | 1.19 |
| 98 | 1pog | 62 | 0.78 | 1.49 | 0.77 | 1.56 | 0.62 | 1.00 | 1.49 | 1.67 | 1.21 |
| 99 | 1pqx | 91 | 0.71 | 1.27 | 0.75 | 0.65 | 0.76 | 1.38 | 1.29 | 1.84 | 1.16 |
| 100 | 1ps2 | 60 | 0.84 | 2.17 | 0.87 | 1.69 | 0.71 | 1.37 | 2.67 | 3.27 | 2.56 |
| 101 | 1pwj | 89 | 0.85 | 1.13 | 0.59 | 0.77 | 0.73 | 0.85 | 0.86 | 1.58 | 1.00 |
| 102 | 1pyc | 41 | 0.72 | 0.98 | 0.52 | 0.69 | 0.48 | 1.22 | 1.01 | 1.86 | 0.87 |
| 103 | 1q1v | 70 | 0.73 | 1.57 | 0.80 | 2.44 | 0.83 | 1.24 | 1.56 | 1.88 | 1.64 |
| 104 | 1q27 | 171 | 0.88 | 1.57 | 0.81 | 1.15 | 0.78 | 1.76 | 1.55 | 2.32 | 1.62 |
| 105 | 1q56 | 195 | 0.80 | 1.04 | 0.80 | 0.78 | 0.82 | 1.04 | 1.40 | 1.74 | 1.19 |
| 106 | 1q5f | 156 | 0.78 | 0.81 | 0.45 | 1.36 | 0.74 | 1.05 | 1.18 | 1.69 | 1.81 |
| 107 | 1q80 | 174 | 0.68 | 0.51 | 0.25 | 0.64 | 0.45 | 0.57 | 1.10 | 1.22 | 1.07 |
| 108 | 1qu5 | 182 | 0.82 | 4.42 | 0.80 | 3.05 | 0.84 | 2.50 | 3.14 | 2.90 | 2.08 |
| 109 | 1rjt | 73 | 0.79 | 2.57 | 0.57 | 2.23 | 0.75 | 1.66 | 2.31 | 1.95 | 2.42 |
| 110 | 1rq6 | 62 | 0.84 | 0.80 | 0.59 | 1.06 | 0.79 | 0.91 | 1.60 | 1.61 | 1.48 |
| 111 | 1s6n | 115 | 0.77 | 1.20 | 0.79 | 0.87 | 0.75 | 1.00 | 0.59 | 1.57 | 0.99 |
| 112 | 1sgg | 67 | 0.33 | 1.29 | -0.13 | 0.56 | 0.15 | 0.91 | 0.38 | 1.32 | 0.75 |
| 113 | 1slj | 96 | 0.72 | 1.21 | 0.65 | 1.19 | 0.85 | 1.18 | 0.86 | 1.71 | 1.46 |
| 114 | 1sm7 | 109 | 0.77 | 0.97 | 0.70 | 1.26 | 0.64 | 1.14 | 1.57 | 1.87 | 1.25 |
| 115 | 1sro | 76 | 0.54 | 0.84 | 0.69 | 0.96 | 0.80 | 0.79 | 1.01 | 1.40 | 1.05 |
| 116 | 1sxe | 97 | 0.81 | 1.10 | 0.87 | 1.50 | 0.78 | 1.09 | 1.35 | 1.85 | 1.60 |
| 117 | 1tcp | 60 | 0.79 | 0.93 | 0.72 | 0.77 | 0.81 | 0.83 | 1.39 | 1.72 | 1.16 |
| 118 | 1tdp | 111 | 0.69 | 0.67 | 0.66 | 0.76 | 0.85 | 0.54 | 0.93 | 1.24 | 1.29 |
| 119 | 1tfi | 50 | 0.51 | 1.55 | 0.91 | 0.66 | 0.33 | 1.33 | 1.58 | 1.53 | 1.61 |
| 120 | 1uaw | 77 | 0.62 | 0.84 | 0.70 | 0.88 | 0.70 | 0.73 | 0.94 | 1.31 | 0.99 |
| 121 | 1uss | 88 | 0.84 | 1.37 | 0.83 | 1.70 | 0.88 | 1.15 | 2.01 | 2.24 | 1.38 |
| 122 | 1v92 | 46 | 0.51 | 0.71 | 0.76 | 1.07 | 0.36 | 1.03 | 0.75 | 1.27 | 1.23 |
| 123 | 1w9r | 119 | 0.76 | 2.48 | 0.63 | 1.45 | 0.84 | 2.43 | 2.62 | 1.81 | 2.99 |
| 124 | 1waz | 46 | 0.29 | 1.10 | 0.15 | 3.14 | 0.79 | 2.41 | 0.92 | 1.43 | 3.26 |
| 125 | 1wm4 | 142 | 0.82 | 0.85 | 0.68 | 0.93 | 0.69 | 0.85 | 1.35 | 1.82 | 1.23 |
| 126 | 1wvk | 86 | 0.62 | 10.31 | 0.58 | 9.62 | 0.58 | 2.08 | 9.46 | 4.01 | 5.50 |
| 127 | 1xhj | 88 | 0.60 | 2.35 | 0.81 | 2.49 | 0.46 | 0.89 | 2.04 | 1.96 | 1.37 |
| 128 | 1xjs | 147 | 0.70 | 0.85 | 0.73 | 1.04 | 0.80 | 0.97 | 1.29 | 1.58 | 1.34 |
| 129 | 1yvc | 70 | 0.86 | 1.41 | 0.83 | 0.77 | 0.73 | 1.63 | 1.38 | 2.50 | 1.45 |
| 130 | 1ywl | 96 | 0.54 | 1.91 | 0.87 | 0.78 | 0.41 | 2.19 | 1.86 | 2.78 | 1.81 |
| 131 | 1z60 | 59 | 0.87 | 0.64 | 0.62 | 0.56 | 0.72 | 0.72 | 0.97 | 1.46 | 1.06 |
| 132 | 1zgg | 150 | 0.65 | 1.11 | 0.65 | 0.49 | 0.81 | 0.73 | 0.33 | 1.34 | 0.71 |
| 133 | 2bid | 197 | 0.85 | 3.61 | 0.84 | 4.36 | 0.87 | 1.13 | 3.21 | 2.20 | 1.82 |
| 134 | 2fmr | 65 | 0.91 | 0.83 | 0.88 | 0.71 | 0.87 | 0.91 | 0.98 | 1.36 | 0.73 |
| 135 | 2hqi | 72 | 0.73 | 0.82 | 0.03 | 0.64 | 0.22 | 0.82 | 0.52 | 1.06 | 0.84 |
| 136 | 2pac | 82 | 0.53 | 1.38 | 0.65 | 0.65 | 0.78 | 1.05 | 0.77 | 1.82 | 0.99 |
| 137 | 2rel | 57 | 0.86 | 1.30 | 0.75 | 2.32 | 0.75 | 1.75 | 2.14 | 3.00 | 3.89 |
| 138 | 2rgf | 97 | 0.49 | 2.69 | 0.23 | 2.86 | 0.67 | 0.82 | 0.49 | 1.94 | 1.50 |
| 139 | 3crd | 100 | 0.75 | 0.55 | -0.02 | 1.30 | 0.30 | 1.03 | 0.89 | 1.28 | 1.63 |
| 140 | 3ifb | 131 | 0.75 | 0.87 | 0.66 | 0.49 | 0.69 | 0.78 | 0.79 | 1.44 | 1.01 |
| **AVERAGE** | | | **0.72** | **1.81** | **0.64** | **1.77** | **0.67** | **1.36** | **1.68** | **1.91** | **1.65** |

**Figure S1.** The graphics below show fluctuation profiles for the entire benchmark set of 140 proteins. The fluctuation profiles present root mean-square fluctuation (RMSF) values (in Angstroms) derived from NMR ensembles (red line) and simulation trajectories: CABS (green line) and MD (blue line). The example profiles from the presented set have been also shown in Figure 1 together with visualizations on 3D models.


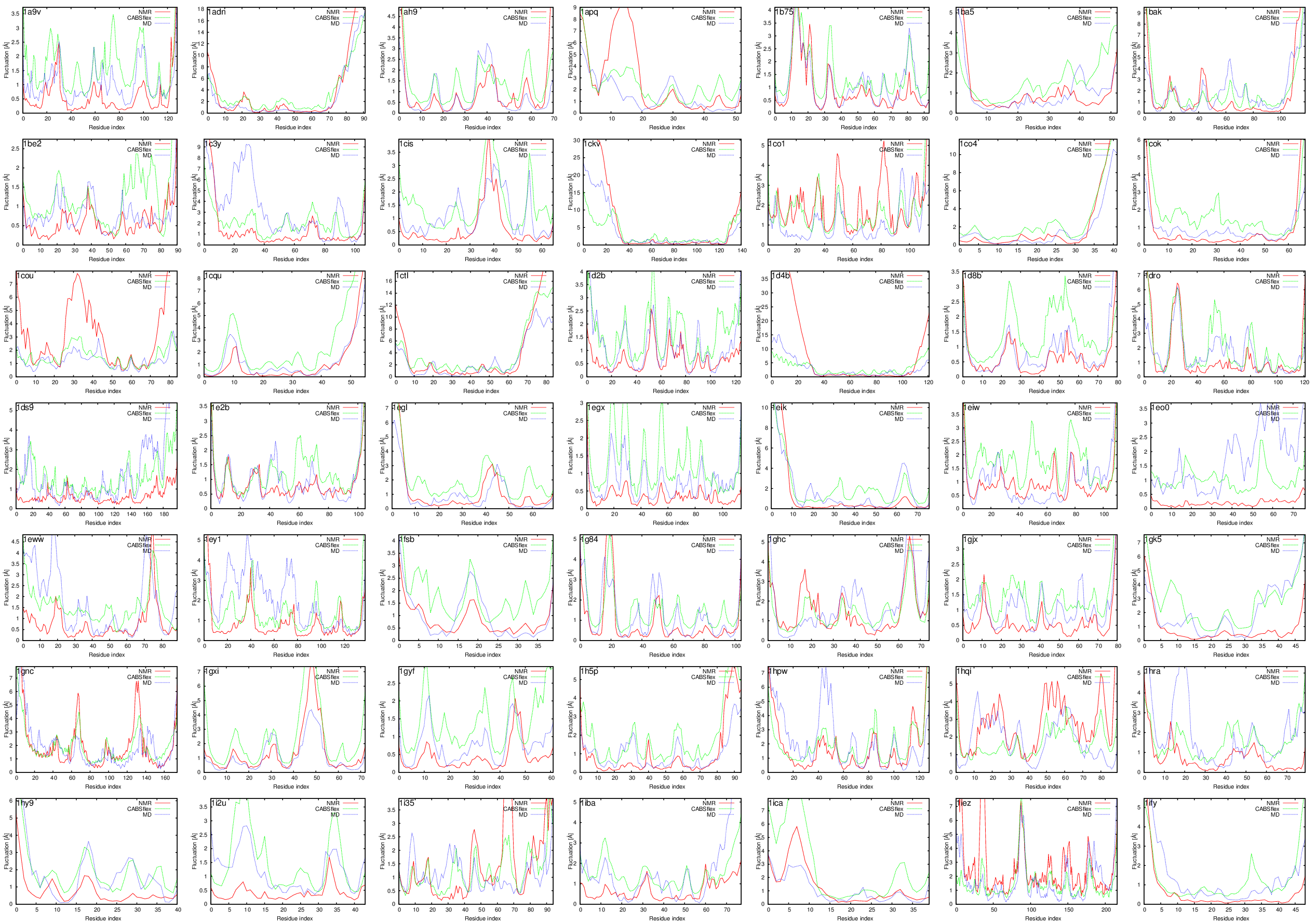


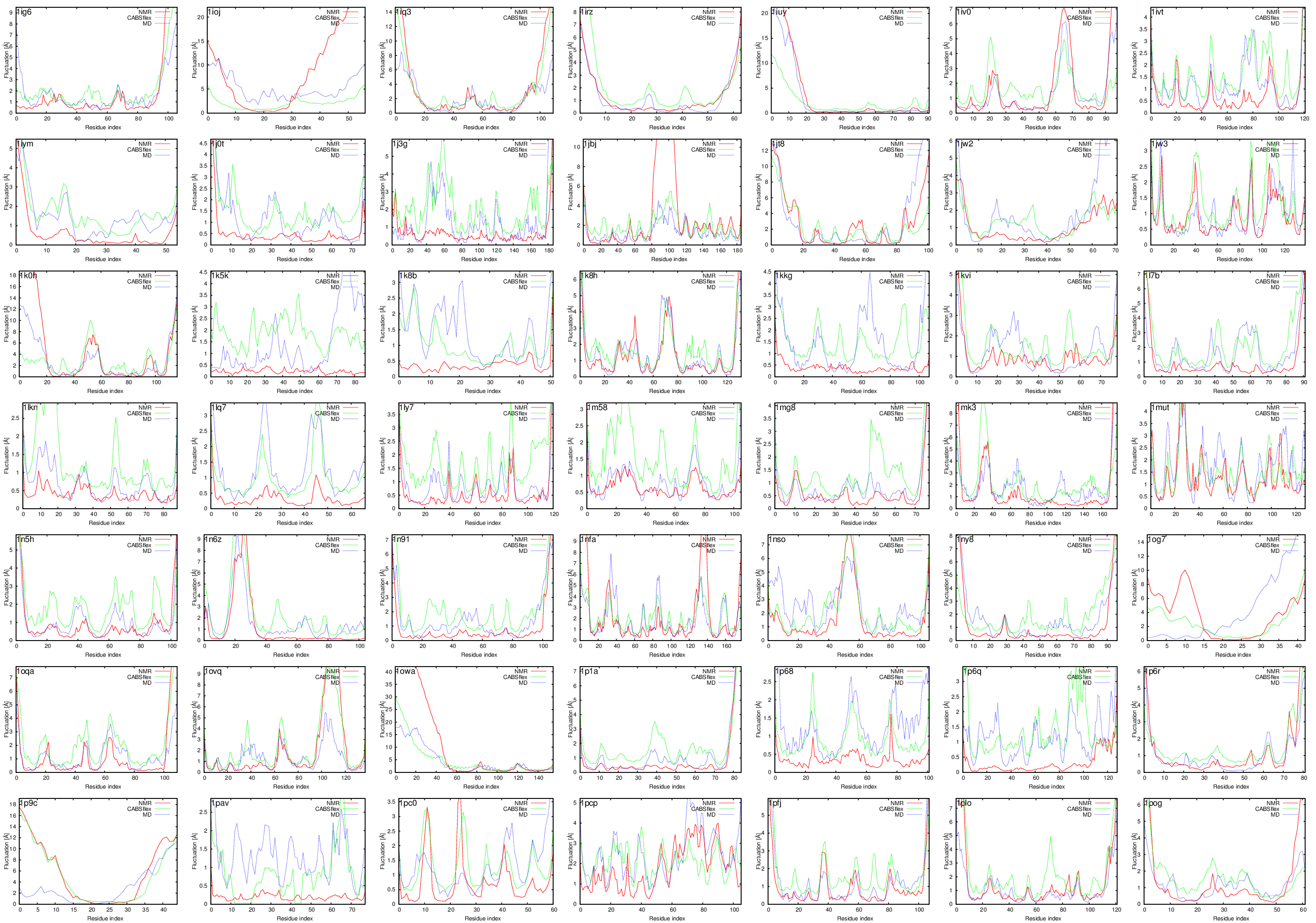


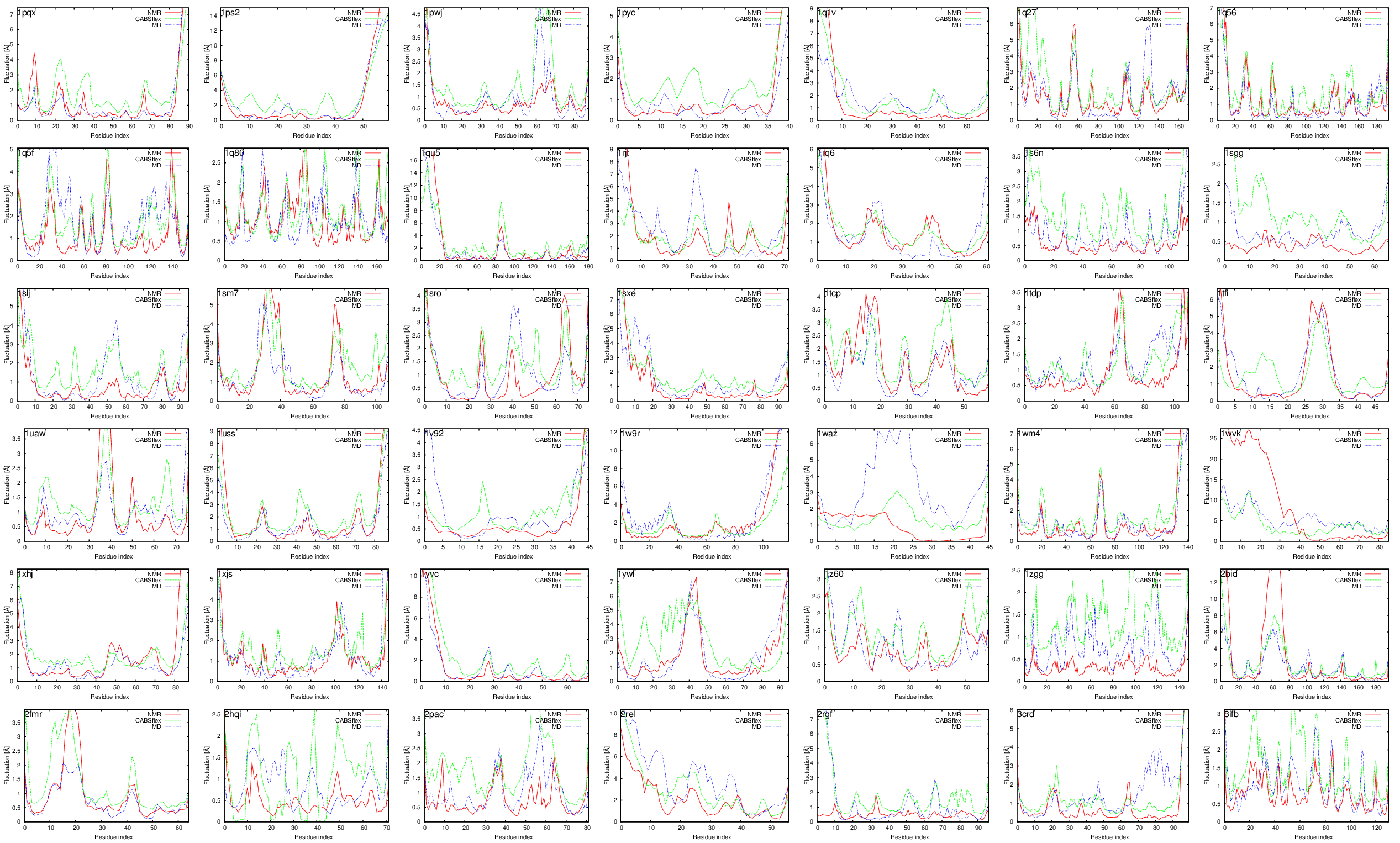

Supplement: Supplementary Data [file supp_btu184_Supplementary_info_rev.docx]
